# Crohn's Disease-Associated Adherent-Invasive *Escherichia coli* Adhesion Is Enhanced by Exposure to the Ubiquitous Dietary Polysaccharide Maltodextrin

**DOI:** 10.1371/journal.pone.0052132

**Published:** 2012-12-12

**Authors:** Kourtney P. Nickerson, Christine McDonald

**Affiliations:** 1 Department of Pathobiology, Lerner Research Institute, Cleveland Clinic, Cleveland, Ohio, United States of America; 2 Department of Molecular Medicine, Cleveland Clinic Lerner College of Medicine, Case Western Reserve University, Cleveland, Ohio, United States of America; Massachusetts General Hospital, United States of America

## Abstract

Crohn's disease (CD) is associated with intestinal dysbiosis evidenced by an altered microbiome forming thick biofilms on the epithelium. Additionally, adherent-invasive *E. coli* (AIEC) strains are frequently isolated from ileal lesions of CD patients indicating a potential role for these strains in disease pathogenesis. The composition and characteristics of the host microbiome are influenced by environmental factors, particularly diet. Polysaccharides added to food as emulsifiers, stabilizers or bulking agents have been linked to bacteria-associated intestinal disorders. The escalating consumption of polysaccharides in Western diets parallels an increased incidence of CD during the latter 20^th^ century. In this study, the effect of a polysaccharide panel on adhesiveness of the CD-associated AIEC strain LF82 was analyzed to determine if these food additives promote disease-associated bacterial phenotypes. Maltodextrin (MDX), a polysaccharide derived from starch hydrolysis, markedly enhanced LF82 specific biofilm formation. Biofilm formation of multiple other *E. coli* strains was also promoted by MDX. MDX-induced *E. coli* biofilm formation was independent of polysaccharide chain length indicating a requirement for MDX metabolism. MDX exposure induced type I pili expression, which was required for MDX-enhanced biofilm formation. MDX also increased bacterial adhesion to human intestinal epithelial cell monolayers in a mechanism dependent on type 1 pili and independent of the cellular receptor CEACAM6, suggesting a novel mechanism of epithelial cell adhesion. Analysis of mucosa-associated bacteria from individuals with and without CD showed increased prevalence of *malX*, a gene essential for MDX metabolism, uniquely in the ileum of CD patients. These findings demonstrate that the ubiquitous dietary component MDX enhances *E. coli* adhesion and suggests a mechanism by which Western diets rich in specific polysaccharides may promote dysbiosis of gut microbes and contribute to disease susceptibility.

## Introduction

Crohn's disease (CD) is a recurrent, debilitating, chronic inflammatory bowel disease with genetic, bacterial, and environmental factors contributing to disease pathogenesis [1]. Extensive genetic studies have expanded our knowledge of predisposing factors, but indicate other risk factors are also important for CD development [2]. Multiple studies indicate that bacteria are central to the onset and perpetuation of CD, as well as demonstrate alterations in the gut bacteria of individuals with CD [3]. However, growing evidence indicates that environmental factors, such as smoking, diet and geography, may play key roles in disease development. Current disease models hypothesize that genetically susceptible individuals develop abnormal immune responses to bacteria in response to environmental stimuli, resulting in inflammatory bowel disease. Currently, it is unclear how environmental factors contribute to the development of disease.

Analyses of the bacterial communities associated with the intestinal mucosa or present in fecal samples of CD patients have identified specific alterations of the microbiome [3]. These alterations include a decrease in microbial diversity, reduced representation of *Firmicutes* and increased levels of *Proteobacteria* [4], [5]. In particular, a lower amount of *Faecalibacterium prausnitzii* and a higher prevalence of *Escherichia coli* have been observed in the ileum of CD patients [3], [6], [7], [8], [9], [10], [11]. These disease-associated *E. coli* have unique properties and are termed “adherent and invasive *E. coli*” (AIEC) for their ability to adhere and invade epithelial cells, as well as survive within macrophages. Interestingly, these strains do not possess any of the classical virulence factor clusters common to other pathogenic *E. coli* strains [12].

Another striking change in the microbiota of CD patients is in the spatial organization of the microbiome. In healthy individuals, the microbiota is separated from direct contact with the intestinal epithelium, while in CD patients gut bacteria form a dense biofilm structure in intimate proximity with the epithelium [13], [14], [15]. The factors which induce this dysbiosis are unclear, but genetics, lifestyle and a “Western” diet (a diet high in fats, sugar and protein but low in fiber) are all proposed to play a role [16].

**Figure 1:**
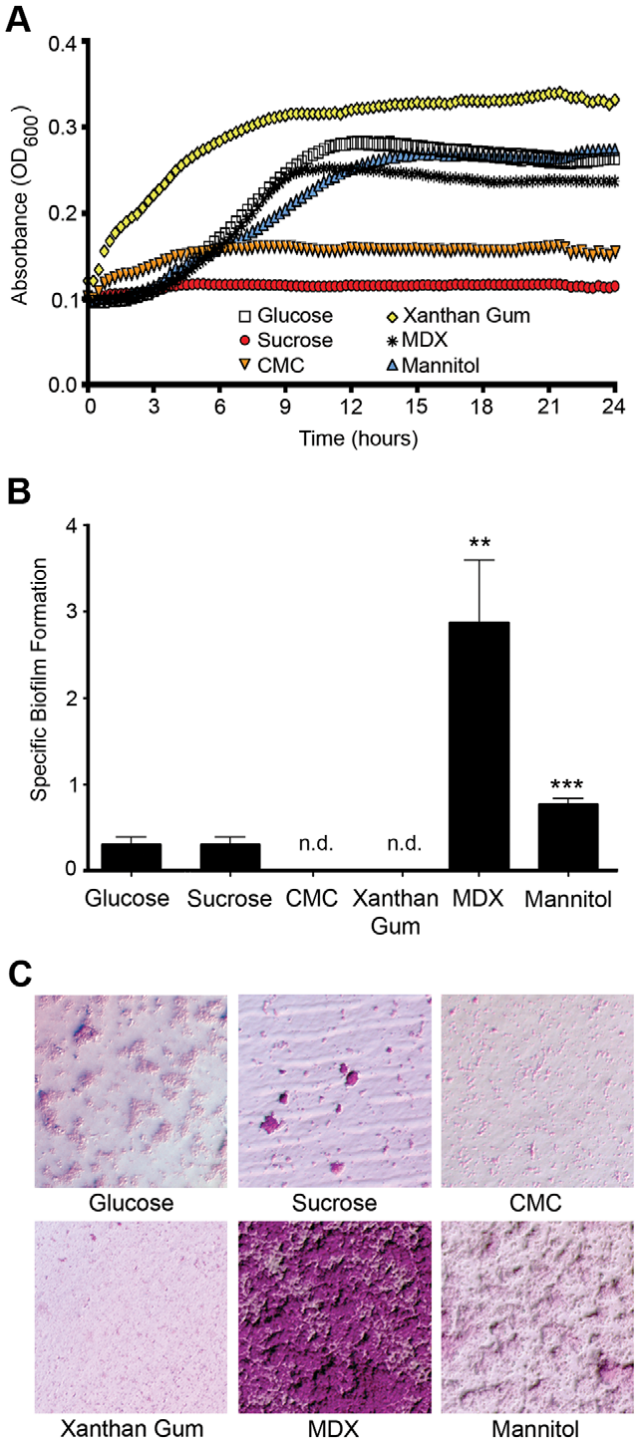
MDX strongly enhances *E. coli* biofilm formation. (A) Growth curves of LF82 grown in medium supplemented with the indicated polysaccharide or sugar. (B) Specific biofilm formation of LF82. Average ±SD shown. **p<0.01, ***p<0.001, n.d. = none detected (C) Micrographs of LF82 biofilms from B stained with Congo red to detect exopolysaccharide formation (pink) with bacteria counterstained with carbol fusion (blue).

Epidemiological studies show a striking increase in the incidence of CD since the 1950 s in the United States [1], [17]. The reason for this explosion is unidentified, but data strongly support an environmental cause. During this time, the availability and popularity of pre-packaged foods escalated in the American diet [18]. To improve the palatability and shelf life of packaged foods, polysaccharides are commonly added as emulsifiers, stabilizers, coating materials or bulking agents. These polysaccharides are generally recognized as safe (GRAS) by the Food and Drug Administration (FDA) and include additives such as carboxymethyl cellulose (CMC), modified starches, carrageenan, pectin, and xanthan gum. However, a growing number of studies link these polysaccharides to intestinal disorders in both animals and humans [19], [20], [21], [22], [23], [24]. Most recently reported is the association of necrotizing enterocolitis with ingestion of xanthan gum by preterm infants [21]. Many of these studies also demonstrate that polysaccharide additives alter the intestinal microbiota, both in the types and amounts of bacteria present.

**Table 1:** Bacterial strains.

Strain	Characteristics	Source or Reference
W3110	K-12 derivative, weak biofilm former	ATCC
MG1655	K-12 derivative, strong biofilm former	ATCC
ECG023	Non-IBD control isolate, Serotype ONT:H-, Phylotype At, *iucD, fimH, fimAvMT*78, weak biofilm former	[55], [56]
ECG043	Non-IBD control isolate, Serotype O83:H1, Phylotype B2, *ibeA, fimH, fimAvMT*78, strong biofilm former	[55], [56]
AIEC19	Non-IBD AIEC isolate, Serotype ONT:H-, Phylotype A, *iucD, fimH, fimAvMT*78, weak biofilm former	[55], [56]
AIEC07	Non-IBD AIEC isolate, Serotype O22:H7, Phylotype B1, *papC, iucD, fimH,* strong biofilm former	[55], [56]
LF82	AIEC isolated from an ileal lesion of a CD patient, Serotype O83:H1	[11]
LF82Δ*fliC*	Isogenic LF82 with *fliC* deletion, KanR	[57]
LF82Δ*fimH*	Isogenic LF82 with *fimH* deletion, KanR	[58]
Nissle 1917	Fecal isolate, Mutaflor® probiotic	Ardeypharm

In light of evidence demonstrating that the composition of the gastrointestinal microbiota is responsive to changes in diet [25], [26], [27], [28] and the strong link between microbial dysbiosis and CD, we investigated whether a polysaccharide dietary additive, such as xanthan gum, CMC, mannitol, or modified starch, could promote CD-associated alterations in *E. coli* adhesion. The results of these experiments demonstrated that the modified starch, maltodextrin (MDX), markedly enhanced *E. coli* biofilm formation and epithelial cell adhesion. MDX is a polysaccharide comprised of α(1→4) and α(1→6) linked chains of 3–20 glucose units produced through the chemical and enzymatic hydrolysis of starch [29]. Our findings demonstrate that MDX alters bacterial adhesion and support a model by which diet influences microbial phenotypes and may contribute to disease development.

**Figure 2:**
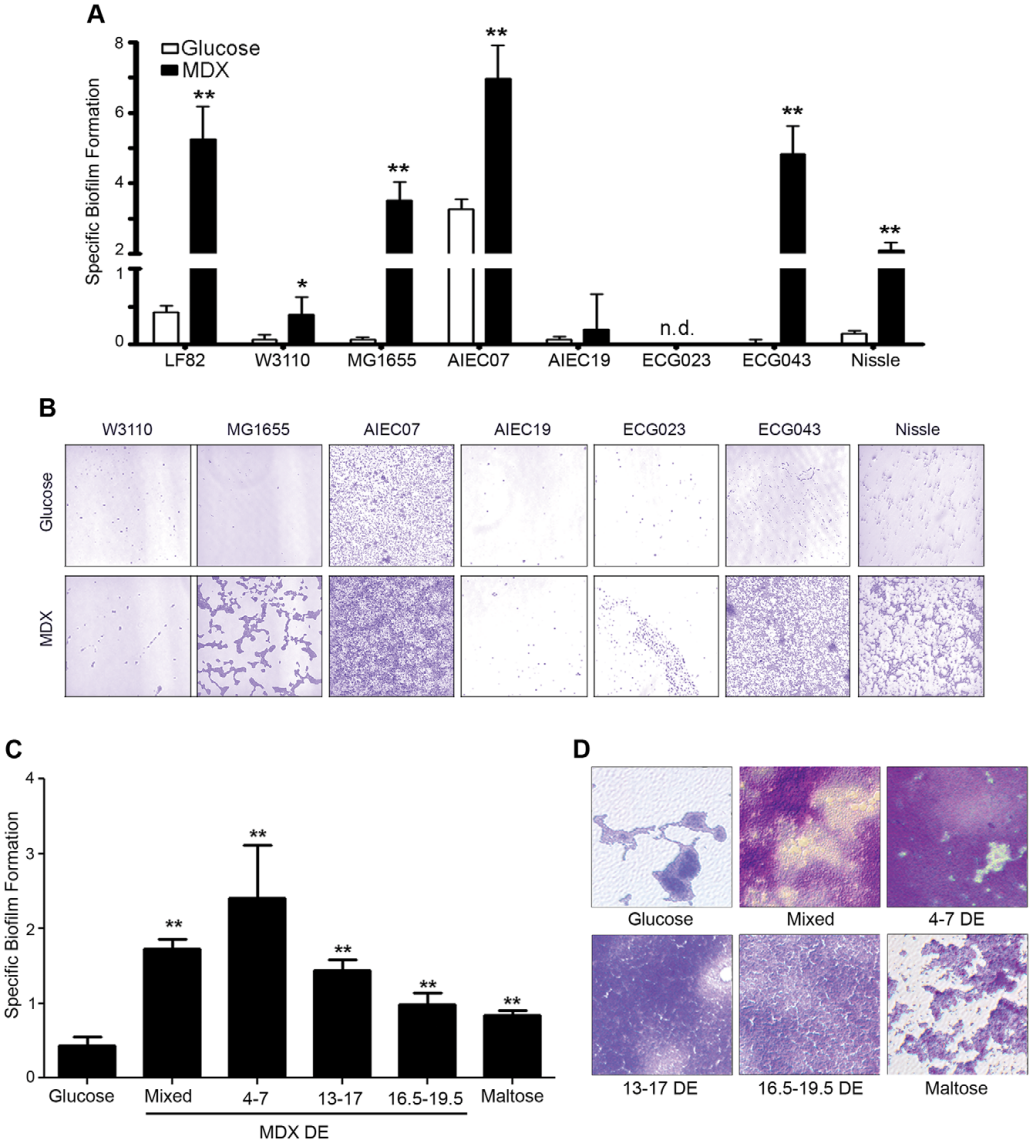
MDX promotes biofilm formation of multiple *E. coli* strains in a process dependent on MDX metabolism. (A) Specific biofilm formation of a panel of *E. coli* strains. (B) Micrographs of crystal violet stained biofilms from A. (C) Specific biofilm formation of LF82 in medium supplemented with MDX of different chain lengths. (D) Micrographs of crystal violet stained biofilms from C. Average ±SD shown. *p<0.05, **p<0.01, n.d. = none detected.

## Materials and Methods

### Ethics Statement

The protocol (IRB#12–383) used to collect the human tissue samples used in this study was reviewed and approved by the Cleveland Clinic Institutional Review Board. This board determined that the collection and anonymous analysis of these de-identified, redundant surgical specimens was exempt from the requirement to obtain informed consent.

**Figure 3:**
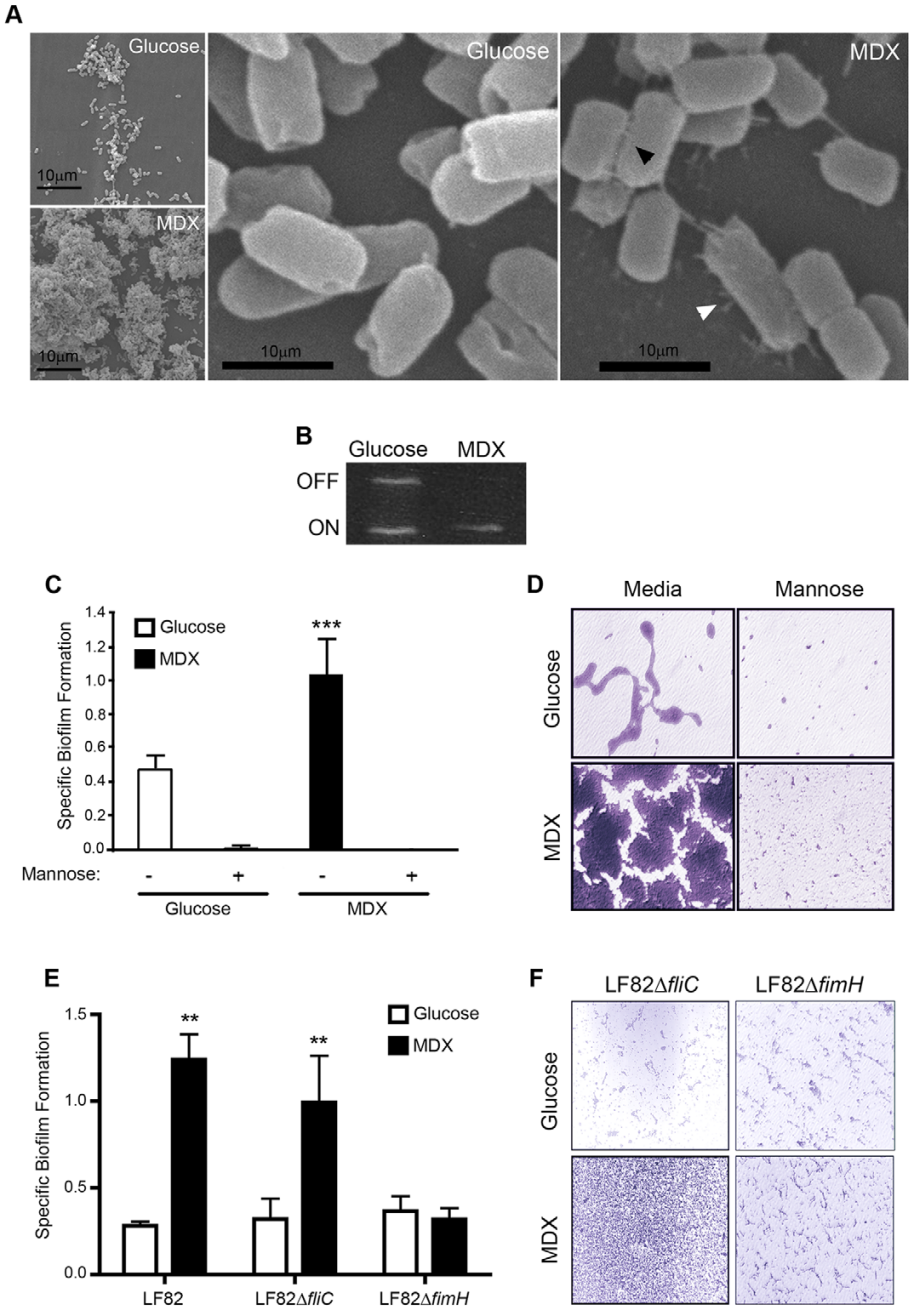
MDX increases biofilm formation via type 1 pili. (A) Scanning electron micrographs of LF82 biofilms. Arrowheads indicate bacteria-plastic (white) or inter-bacterial (black) adhesins. (B) Assessment of the LF82 *fim* operon by PCR to determine type 1 pili expression. (C) Specific biofilm formation of LF82 in the presence or absence of 2% mannose. (D) Micrographs of crystal violet stained biofilms from C. (E) Specific biofilm formation of LF82 and isogenic mutant strains. (F) Micrographs of crystal violet stained biofilms from E. Average ±SD shown. *p<0.05, **p<0.01.

### Patient Samples and Quantitative Real Time PCR

De-identified gastrointestinal tissue was collected from ileal and colonic resections by the Cleveland Clinic Tissue Procurement Service (IRB#12–383; Table S1). DNA was isolated from unblotted tissue using the Roche High Pure PCR Template Prep Kit for genomic DNA isolation. Real-time quantitative PCR (qPCR) was performed using 10ng DNA and primers for *malX (*5′ACGCGTTTCCTTTCGCAA3′/5′ACAGAACTGGCGCTACGA3′), *E. coli* 16 S (5′CATGCCGCGTGTATGAAGAA3′/5′CGGGTAACGTCAATGAGCAAA3′) [30] or *Eubacteria* (5′TCCTACGGGAGGCAGCAGT3′/5′GGACTACCAGGGTATCTAATCCTGTT3′) [31] in iTaq SYBR Green Supermix with ROX (Biorad) on an ABI prism 7900HT using SDS2.4 software. Samples were run in triplicate with *E. coli 16* *S* and *malX* values normalized to *Eubacteria* levels using the equation: 2ˆ-(CT_X_ – CT_eub_).

**Figure 4:**
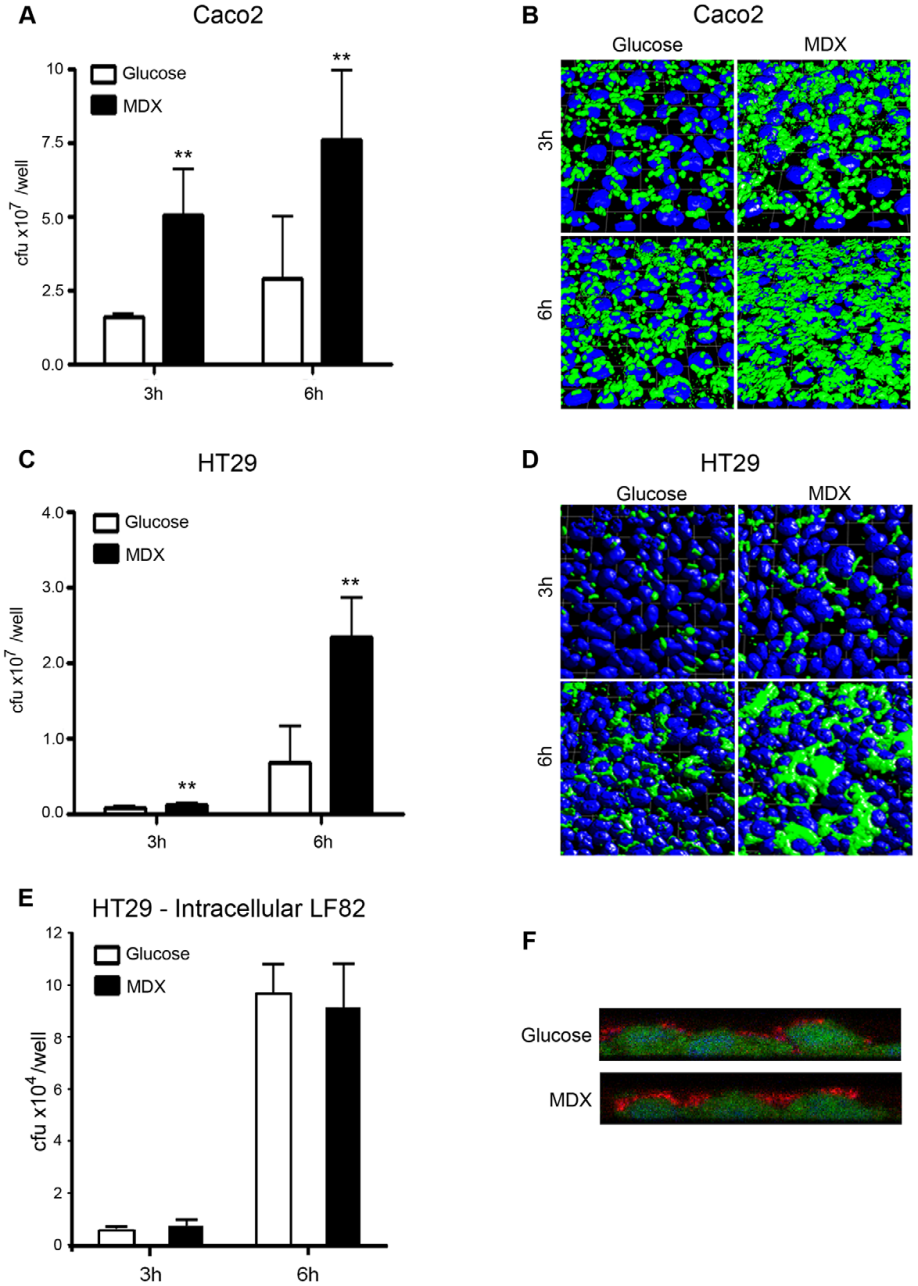
MDX selectively enhances LF82 adhesion to intestinal epithelial cell lines. (A) Adhesion of LF82 to Caco2 monolayers. (B) Immunofluorescent confocal micrographs of LF82 adhered to Caco2 monolayers. Green = LF82, blue = nuclei (C) Adhesion of LF82 to HT29 monolayers. (D) Immunofluorescent confocal micrographs of LF82 adhered to HT29 monolayers. Green = LF82, blue = nuclei (E) Intracellular LF82 recovered from HT29 monolayers. (F) Immunofluorescent confocal micrographs demonstrating the localization of LF82 (red) on the surface of HT29 cells (green). Nuclei  =  blue. Average ±SD shown. *p<0.05, **p<0.01 relative to glucose.

**Figure 5:**
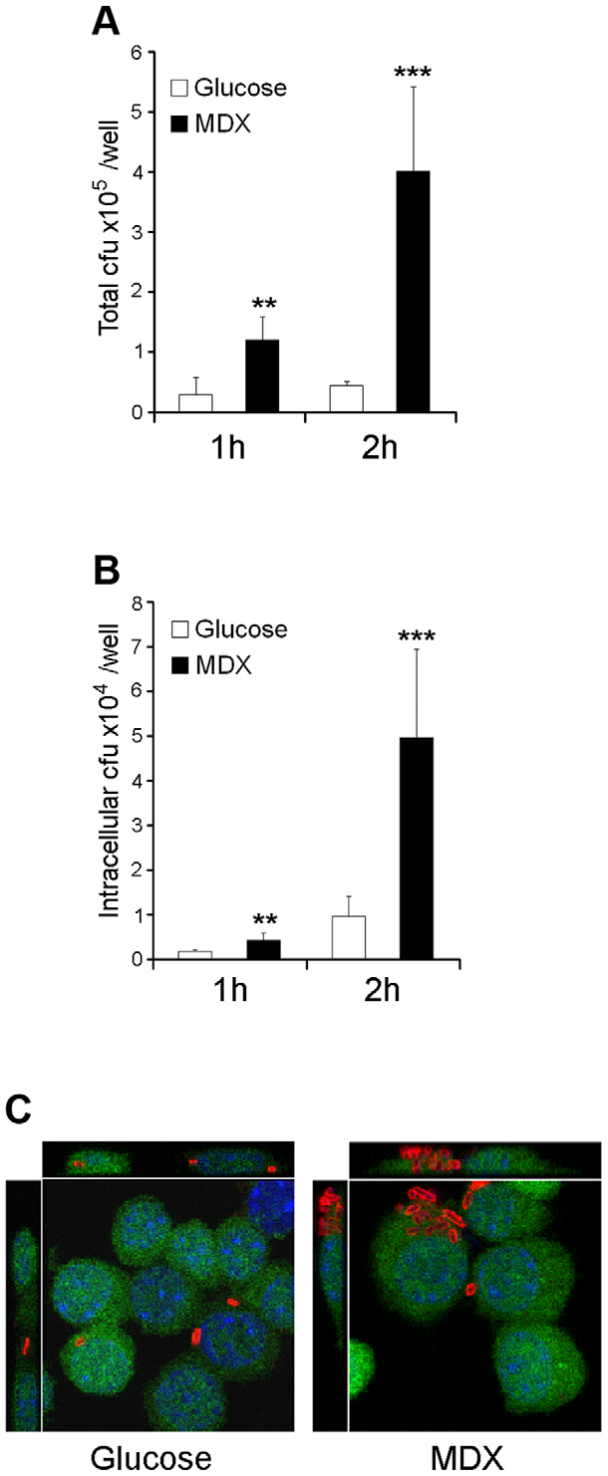
MDX selectively enhances LF82 adhesion to Raw264.7 macrophages. (A) Total amount of Raw264.7 cell-associated LF82. (B) Intracellular LF82 recovered from Raw264.7 cells. (C) Immunofluorescent confocal micrographs of LF82 (red) infected Raw264.7 cells (green). Nuclei  =  blue. Average ±SD shown. **p<0.01, ***p<0.001 relative to glucose.

### Biofilm formation assays

Specific biofilm formation assays were adapted from a microtiter plate protocol [32]. Briefly, M9 minimal medium (M9 Salts (BD Difco), 2 mM MgSO_4_ (Acros), 100 μM CaCl_2_ (Sigma)) supplemented with 0.4% sugars was prepared fresh the week of use. Glucose (Acros), MDX (Spectrum chemicals), fractionated MDXs, maltose, sucrose, D-mannitol, xanthan gum, cellulose and sucralose were purchased from Sigma. Aspartame was purchased from Supelco. “Measures like sugar” formulations of Splenda® and Equal®, as well as liquid Sweetleaf® stevia were purchased from a grocery store in Cleveland, Ohio. The final concentration of supplemental sugar (0.4% w/v) added to the minimal media was based on the estimated volume of the stomach (1L) and the serving size (0.5 g) of the commercial products, Splenda® and Equal®. Overnight cultures of bacteria were grown in LB broth at 37°C without shaking. M9 medium was inoculated 1∶100 and plated in triplicate in 96 well plates then incubated at 30°C for 24 h. The OD_600_ of each well was measured, then wells were washed with phosphate buffered saline (PBS) and air dried for >20 min. Wells were stained for 5 min with 1% crystal violet, washed 4x dH_2_O and air-dried overnight in the dark. Plates imaged using an Olympus 1X71 microscope with a 40x LucPlanFLN RC3 objective with complementary RC3 filter and a RTKE Diagnostic SPOT camera. Wells were then de-stained in 95% ethanol, OD_540_ measured and specific biofilm formation was calculated using the following equation: (OD_540_ - medium OD_540_)/OD_600_. The exopolysaccharide matrix of 4% paraformaldehyde/PBS fixed biofilms was stained with saturated Congo red overnight and bacteria counterstained with carbol fuscin. Growth curves were determined by measuring the OD_600_ every 15 min.

**Figure 6:**
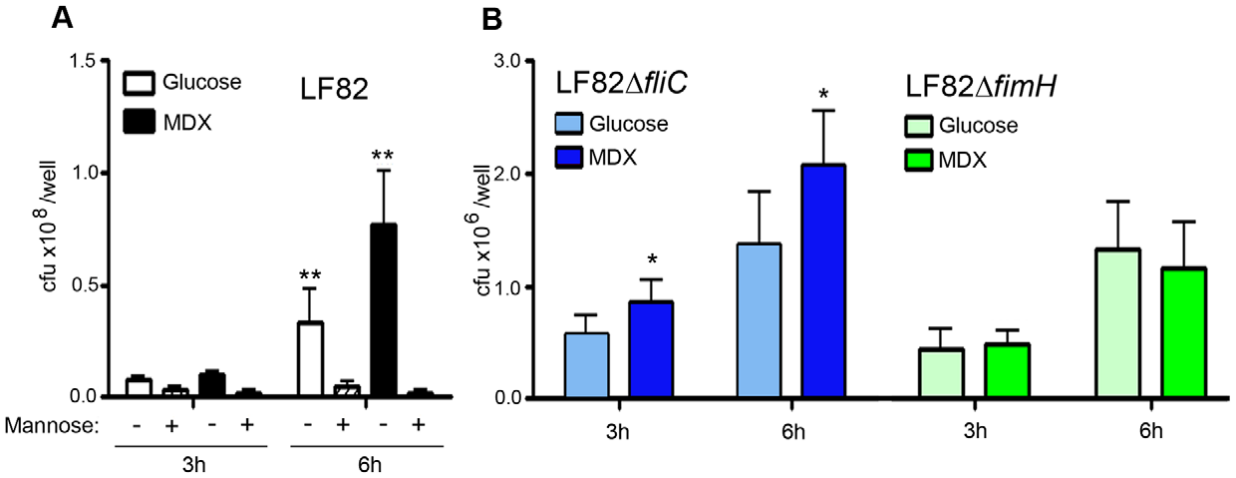
MDX enhances epithelial cell adhesion in a type 1 pili-dependent manner. (A) Adhesion of LF82 to HT29 monolayers pre-incubated with 2% mannose. (B) Adhesion of LF82 isogenic mutants to HT29 monolayers. Average ±SD shown. *p<0.05, **p<0.01 relative to glucose.

### Scanning Electron Microscopy

Biofilms were grown on Thermanox® plastic coverslips (Nunc) for 24 h at 30°C. Samples were washed with PBS and fixed in 4% paraformaldehyde (PF)/2.5% glutaraldehyde/PBS overnight at 4°C. After washing in PBS, samples were placed in 1% osmium tetroxide for 1 h. Samples were washed with dH2O for 5 minutes, followed by dehydration in a graded series of ethanol (50%, 70%, 95% EtOH) with 3×10 minutes in absolute EtOH. Samples were dried 2×10 min in 100% EtOH:hexamethyldisilazane, 1∶1 and 10 min in 100% hexamethyldisilazane x3 at RT. Samples were gold coated and examined with a JEOL Scanning Microscope (JSM 5310).

**Figure 7:**
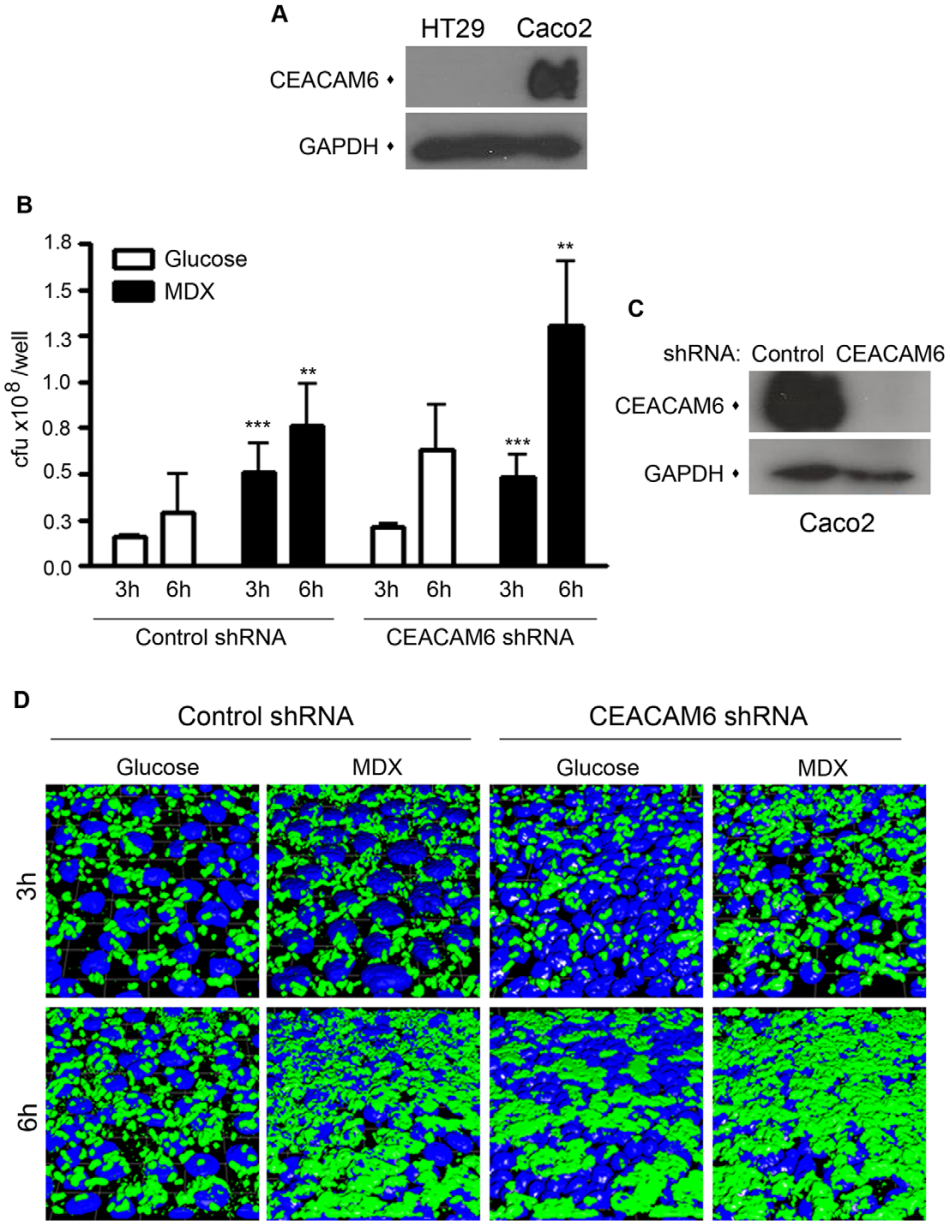
MDX-enhanced LF82 adhesion to epithelial cells occurs via a mechanism independent of CEACAM6. (A) Immunoblots of CEACAM6 expression. (B) Adhesion of LF82 to Caco2 cell lines stably expressing shRNAs. Average ±SD shown. **p<0.01, ***p<0.001 relative to glucose. (C) Immunoblots of CEACAM6 expression. (D) Immunoflurescent confocal micrographs of LF82 adhered to Caco2 cells used in B. Green = LF82, blue = nuclei.

### Type 1 Pili Expression Analysis

For analysis of the *fim* operon, biofilms were lysed using Roche High Pure PCR Template Preparation Kit for isolation of genomic DNA. Analysis of the *fim* operon was performed by PCR amplification of the *fimA/E* invertible element as described [33].

**Figure 8:**
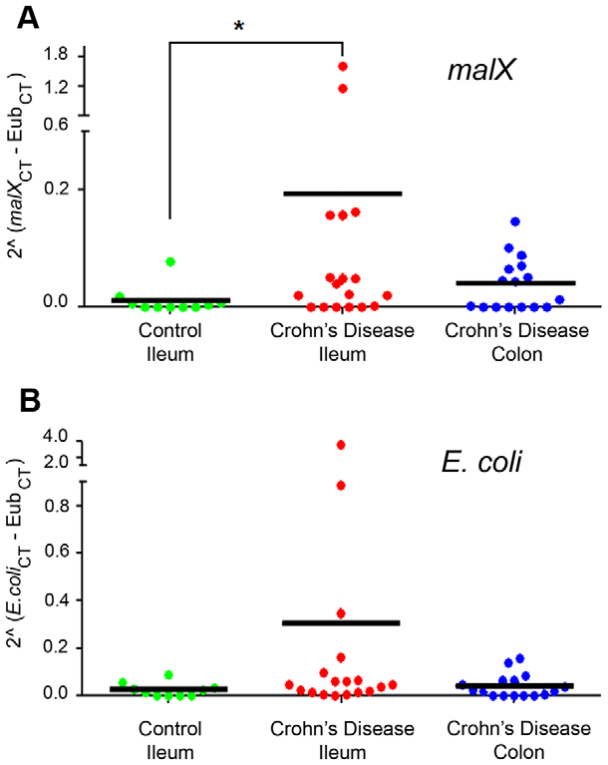
Bacteria with the *malX* gene are more prevalent in the mucosa of ileal CD. (A) Prevalence of the *malX* gene normalized to total *Eubacterial* DNA (*Eub*) amplified from mucosal samples by qPCR. (B) Prevalence of *E. coli* 16S DNA (*E. coli*) as measured in A. Mean indicated with bar. *p<0.0175.

### Cell culture

HT29 cells (American Type Culture Collection (ATCC); gift of Carol de la Motte, Cleveland Clinic) were maintained in RPMI with 10% fetal bovine serum (FBS; Invitrogen). Caco2 cells (ATCC; gift of Lopamura Das, Case Western Reserve University) were maintained in DMEM with 20%FBS. Raw264.7 cells (ATCC; gift of Gabriel Nuñez, University of Michigan) were grown in DMEM with 10%FBS. Caco2:shCeacam6 and Caco2:shControl stable knockdown cell lines were generated by infection with MISSION shRNA lentiviruses (SHC002 and NM_002483.3–222 s1c1, Sigma) followed by puromycin selection.

### Cell adhesion assays

Infections of epithelial cell monolayers (multiplicity of infection (MOI) = 10) were performed with 15–21d cultures of Caco2 cell lines, 5d cultures of HT29 cells or Raw264.7 cells plated the previous day. *E. coli* cultures were diluted in culture media for infection. For epithelial cell infections, wells were washed 2x PBS after 3 h, and cells were harvested or media replaced for an additional 3 h. For Raw264.7 cell infections, cells were washed 2x PBS and harvested 1 h or 2 h post-infection. At harvest, wells were washed 2x PBS, lysed in 0.1% Triton X-100/PBS (epithelial cells) or 1% Triton X-100/PBS (Raw264.7) and plated on LB plates in duplicate for calculation of colony forming units/well.

### Cell invasion assays

HT29 monolayers and Raw264.7 cells were infected at an MOI of 10. After 2.5 h and 5.5 h infection of HT29 monolayers or 0.5 h and 1.5 h post-infection of Raw264.7 cells, cells were washed 2x PBS and media containing 100 μg/mL gentamycin was added to the wells. Cells were incubated for an additional 30 min, then cells were lysed in 0.1% Triton X-100/PBS (HT29) or 1% Triton X-100/PBS (Raw264.7) and plated on LB agar in duplicate for calculation of colony forming units/well.

### Immunofluorescence

Cells infected with LF82 on glass coverslips were fixed in 4%PF/PBS (Electron Microscopy Services) then permeabilized with 0.4% Triton X-100/PBS, blocked in 2%FBS/PBS and stained with anti-*E.coli* antibody (ab20640, Abcam). Coverslips were incubated with goat anti-rabbit-Alexa488 (Invitrogen) in 2%FBS/PBS and mounted on slides with Vectashield+DAPI (Vector Labs). For visualization of intracellular LF82, cell cytoplasm was visualized by addition of Cell Tracker CMFDA (1 μM final concentration, Molecular Probes) during infection. Cells were infected for 45 min, then washed 2x PBS and then incubated for an additional 30 min in media containing 100 μg/mL gentamycin. Coverslips were stained for *E. coli* as described above. Confocal microscopy performed using a Leica TCS-SP spectral laser scanning confocal microscope equipped with a Q-Imaging Retiga EXi cooled CCD camera and Image ProPlus Capture and Analysis software (Media Cybernetics). The *z*-stacks (0.5 μM step, line average of 4) were imported into Volocity (version 6.0.1) and analyzed using ‘isosurface’ with 1–4% black and 1–2x brightness.

### Statistical Analyses

Figures are representative outcomes of a minimum of three independent experiments. Experiments were performed in triplicate and significance determined by ANOVA with post-hoc analysis using unpaired t-tests with equal variance. qPCR data was analyzed by non-parametric Wilcoxon test to accommodate the non-Gaussian distribution of the data set.

## Results

### Maltodextrin enhances biofilm formation of CD-associated *E. coli in vitro*

We investigated whether specific polysaccharides used as emulsifiers, thickeners, coating agents or stabilizers in processed foods influenced growth or adhesive characteristics of an *E. coli* strain associated with Crohn's disease, AIEC LF82. The growth of LF82 in minimal medium supplemented with CMC, xanthan gum, MDX, or mannitol was assessed in comparison to sucrose or glucose over 24 hours. LF82 grew in all media formulations with the exception of media supplemented with CMC or sucrose (Figure 1A).

As microbial communities in the intestine form biofilm structures with characteristics distinct from actively growing planktonic cultures, the effect of the polysaccharides on LF82 specific biofilm formation on plastic was measured after 24 hours. Specific biofilm formation was strikingly enhanced in medium containing MDX relative to glucose-supplemented medium and more modestly increased in mannitol-containing medium (Figure 1B). This is in contrast to LF82 growth in medium supplemented with CMC or xanthan gum which did not form detectable biofilms. Media with sucrose did not significantly alter LF82 biofilm formation in comparison to glucose-supplemented medium biofilms. Biofilm formation was confirmed by microscopy in wells stained with Congo red to visualize exopolysaccharide matrix production, a hallmark of biofilm formation (Figure 1C). These results indicate that MDX, a polysaccharide derived from starch hydrolysis, markedly promotes biofilm formation of the CD-associated LF82 *E. coli* strain.

MDX is included as a bulking agent in the no-calorie sweeteners Equal® (aspartame) and Splenda® (sucralose). Using these commercial sources of MDX, the growth and biofilm formation of LF82 was assessed. LF82 grew robustly in media supplemented with Equal® or Splenda® (Figure S1A) and specific biofilm formation was strikingly enhanced in medium containing Equal® or Splenda® relative to glucose-supplemented medium (Figures S1B and S1C). The effect of replacing MDX with glucose as a filler component for aspartame or sucralose was also tested in specific biofilm formation assays. No increases in biofilm formation were observed in glucose-containing medium supplemented with aspartame or sucralose (Figures S1D and S1E). Likewise, addition of the artificial sweeteners to MDX-containing medium did not further increase biofilm formation over the levels observed in medium supplemented only with MDX. These findings indicate that MDX found in commercial sources can stimulate LF82 biofilm formation.

### MDX promotes biofilm formation of multiple *E. coli* strains in a process dependent on MDX metabolism

MDX is utilized by bacteria through a conserved bacterial maltose/MDX metabolic system [34] suggesting that MDX enhancement of biofilm formation may be a general characteristic of *E. coli*. A panel of *E. coli* strains including laboratory reference strains, additional AIEC strains, clinical isolates from individuals without inflammatory bowel disease and the probiotic *E. coli* Nissle 1917 (Table 1) were evaluated for growth and biofilm formation in medium supplemented with glucose or MDX. A majority of the strains tested (75%) showed a significant increase in MDX-stimulated specific biofilm formation relative to glucose-supplemented medium (Figure 1E and 1F). These findings suggest that MDX affects a wide variety of *E. coli* strains and that this is not a unique feature of disease-associated strains.

MDX is a glucose polymer between 3–20 glucose units. The length of these polymers is defined by dextrose equivalent (DE), with longer chains having lower DE values [29]. In studies of other carbohydrates, it has been demonstrated that chain length dramatically alters the biological effect of the carbohydrate polymers [35]. Since the MDX tested is a heterogeneous mix of chain lengths, the effectiveness of different size MDXs to stimulate biofilm formation was investigated to determine whether a specific size range of MDX was required for this effect. All MDX-supplemented media, regardless of chain length, were sufficient to increase LF82 biofilm formation relative to glucose-supplemented medium in specific biofilm formation assays and confirmed by staining of biofilms with crystal violet or Congo red (Figures 2C, 2D and Figure S2). While all MDX-supplemented media promoted biofilm formation, longer MDX chains were more effective, suggesting that metabolism of MDX may be important for biofilm enhancement.

Addition of long polymers to solutions can alter osmolarity. Biofilm formation and LF82 adhesion to epithelial cells have been demonstrated to be influenced by changes in osmolarity [36], [37]. To evaluate whether MDX supplementation altered the osmotic strength of the medium, we measured the osmolarity of the media used in our assays by freezing-point osmometry. Minor changes in osmolarity (less than 20 mOsm/kg) were observed in medium supplemented with either the heterogeneous mix of DEs (Mixed) or 4–7 DE MDX, while no change in osmolarity was detected in medium containing either 13–17 DE or 16.5–19.5 DE MDXs (data not shown). However, all MDX-containing media promoted biofilm formation, indicating that osmolarity changes are not required for MDX to induce biofilm formation of *E. coli*.

### MDX promotes type 1 pili expression which is required for enhanced biofilm formation

To identify candidate bacterial adhesins responsible for MDX-mediated biofilm formation, LF82 biofilms were visualized by scanning electron microscopy (SEM). At low magnification, we observed enhanced adherence of LF82 in MDX-containing medium to the coverslip, as well as to other bacteria (Figure 3A). At higher magnification, an increase in short, thin, hair-like projections ranging between 0.5 μm to 1.5 μm in length could be seen protruding from the surface of LF82 grown in medium supplemented with MDX. One adhesin with these characteristics is type 1 pili, which has been described as a major adhesive structure of LF82 [10], [38]. Expression of type 1 pili is regulated by an invertible DNA element in the *fim* operon of the bacterial genome. To confirm the expression of type 1 pili by MDX, we assessed the position of the *fim* operon of LF82 in 24 h biofilms by PCR (Figure 3B). The adherent bacteria in glucose-containing medium were found to be a mixed population of type 1 pili expressors and non-expressors, similar to what was observed by SEM. In contrast, the bacteria in MDX supplemented medium were more homogenous with the *fim* invertible element in the “on” position, suggesting that type 1 pili are adhesins upregulated by LF82 when grown in MDX-containing medium. Next, we examined whether type 1 pili were functionally required for MDX-enhanced LF82 biofilm formation. Type 1 pili attach to cells and surfaces in a mannose-sensitive manner dependent on the tip protein FimH [10], [38]. Competition with mannose abrogated specific biofilm formation by LF82 in either glucose or MDX supplemented medium (Figures 3C and 3D), indicating that MDX-enhanced biofilm formation is due to an upregulation of a mannose-sensitive adhesin. We confirmed this adhesin to be type 1 pili through the evaluation of isogenic mutant strains of LF82. A type 1 pili adhesin *fimH* knockout strain (Δ*fimH*) formed biofilms that were unaffected by the addition of MDX, whereas a flagellin knockout strain (Δ*fliC*) increased biofilm formation in MDX-containing medium (Figures 3E and 3F). Taken together, this data demonstrates that MDX increases biofilm formation through the upregulation of type 1 pili expression.

### MDX enhances LF82 adhesion to intestinal epithelial cells and macrophages, but does not promote invasion

LF82 has been shown to adhere and invade intestinal epithelial cells [10], [38]. We assessed whether growth in MDX promoted adhesion and invasion of LF82 to human intestinal epithelial cell lines (Caco2 and HT29) and a macrophage cell line (Raw264.7). LF82 were grown overnight in medium supplemented with either glucose or MDX. These bacteria were brought into early log phase of growth in fresh medium for infection. To assess adhesion, the total number of adherent bacteria was quantified after 3 or 6 hours by colony counts and visualized by confocal microscopy. Growth in MDX supplemented media increased the number of LF82 adhered to intestinal epithelial cell monolayers at both timepoints in comparison to LF82 from glucose-containing medium (Figures 4A–D). Similar results were observed in infections of Raw264.7 macrophages, with both quantitative and visual measures of adherence demonstrating increased LF82 adhesion when the bacteria were grown in MDX (Figures 5A and 5C).

In addition to LF82 adhesion, invasion of both HT29 and Raw264.7 cells was assessed by gentamycin protection assay and confocal microscopy. The total number of intracellular LF82 in HT29 epithelial cells was unaffected by MDX (Figures 4E and 4F). In contrast, we observed increased numbers of intracellular MDX grown LF82 in Raw264.7 macrophages (Figures 5B and 5C). The amount of intracellular LF82 was proportional to the number of LF82 adhered to the macrophages. Therefore, we postulate that the increased intracellular load of MDX grown LF82 in the macrophages is likely due to phagocytosis of a higher number of bacteria adhered to the surface of the cells. These findings indicate that MDX has a significant effect on LF82 adhesion to cells but does not increase the invasiveness of this strain.

### LF82 adhesion to intestinal epithelial cells is enhanced by MDX in a type 1 pili dependent manner

LF82 has been shown to adhere to intestinal epithelial cells in a type 1 pili-dependent manner [10], [38]. To determine the role of type 1 pili in the enhanced adhesion of LF82 by MDX, binding of LF82 to cells was competed by addition of mannose during infection, suggesting that LF82 adhesion may be mediated by type 1 pili (Figure 6A). The requirement of type 1 pili for MDX enhancement of LF82 cell adhesion was confirmed by infection studies with the Δ*fliC* and Δ*fimH* LF82 mutant strains. In agreement with previous studies, [10], [39] overall adhesion to epithelial cells of both the Δ*fliC* and Δ*fimH* strains was significantly reduced (∼2 logs) relative to the wild-type strain (Figure 6B). However, while adhesion of the Δ*fliC* mutant strain was enhanced by exposure to MDX, there was no effect of MDX on the adhesion of the Δ*fimH* strain. Together this data demonstrates that the increased type 1 pili expression by LF82 grown in MDX not only enhances biofilm formation, but also increases adhesion to human intestinal epithelial cell monolayers.

### MDX-enhanced LF82 adhesion to intestinal epithelial cells is independent of CEACAM6

Previous studies demonstrate that LF82 adhesion to ileal enterocytes from CD patients and colonization of the mouse intestine requires the binding of type 1 pili to the cellular receptor CEACAM6 [38], [40]. Surprisingly, when we evaluated the expression levels of CEACAM6 by immunoblot of the intestinal cell lines used in our experiments, the HT29 cell line had no detectible CEACAM6 expression (Figure 7A). This suggested that MDX-enhanced adhesion of LF82 was independent of CEACAM6. To confirm this finding, we generated Caco2 cell lines with the endogenous CEACAM6 expression stably knocked down by RNAi (Figure 7C). In infection assays, loss of CEACAM6 expression did not alter the number of either glucose- or MDX-exposed LF82 adhering to Caco2 monolayers, as quantitated by colony counts or visualized by confocal microscopy (Figures 7B and 7D). These findings indicate that MDX promotes adhesion to intestinal epithelial cells via a mechanism independent of CEACAM6.

### Bacteria with genes for MDX metabolism are more prevalent in the ileal mucosa of CD patients

AIEC strains have been isolated from CD patients with ileal disease and are implicated in CD pathogenesis [3], [6], [11]. A recent study observed that these AIEC strains are present at the time of disease diagnosis and these strains were associated with carriage of a virulence factor, *malX* [41]. MalX is a MDX-binding component of the maltose/MDX metabolism system [42]. As MDX-grown LF82 form robust biofilms, we hypothesized that MDX metabolism may be beneficial for colonization of *E. coli* in the terminal ileum of CD patients. We designed real-time quantitative PCR (qPCR) primers for amplification of *malX* to determine if bacteria with MDX metabolism genes were more prevalent in CD patient mucosal samples (Figure S3). DNA samples from intestinal mucosa were analyzed by qPCR for the presence of *malX* and *E. coli* 16S DNA normalized to total levels of *Eubacteria* DNA. The normalization of these genes relative to total *Eubacteria* levels allowed us to determine if the changes we observed were due to population shifts and not to overall increases in *Eubacteria* levels that have been reported in CD patients [43]. Specifically in ileal samples, we observed increased levels and prevalence of the *malX* gene in CD patients as compared to controls (18% positive in ileal controls vs. 71% positive in ileal CD) (Figure 8A). The prevalence of *E. coli* 16S DNA was not increased in these samples, indicating that *malX* is not a marker for *E. coli* (Figure 8B). These findings suggest a link between MDX utilization and microbial changes in ileal CD.

## Discussion

The pivotal contribution of the intestinal microbiome to both health and disease is becoming increasingly appreciated through studies defining this “organ” and how it is altered in disease states [44]. The large microbial community residing in the intestine provides protection against pathogens, aids in digestion and absorption of nutrients, production of micronutrients, neutralization of toxins, and the shaping of the immune system. Alterations in the composition or function of this microbial ecosystem has been demonstrated in diseases such as athlerosclerosis, obesity, metabolic syndrome, allergy, diabetes and inflammatory bowel disease [45]. Therefore, elucidating factors which modulate the microbiota are of importance in understanding disease pathogenesis.

In the case of CD, pathogenesis is linked to intestinal dysbiosis characterized by an increase in total numbers of bacteria, a reduced diversity of bacterial species, and alterations in the spatial organization of the microbiome [3]. Specifically, the concentration of bacteria in biofilms adhered to the intestinal epithelium of CD patients are 2 logs higher than found in controls [15]. Additionally, ileal CD is associated with increased prevalence of *E. coli* strains, including AIEC strains such as LF82 [11]. These AIEC strains have been identified in individuals newly diagnosed with CD [41], but because of their low prevalence (∼6%) in healthy individuals [11] are not thought to be pathogens but rather pathobionts – opportunistic bacteria which under certain circumstances can promote disease [46]. CD is a complex and multi-factorial disease with genetic, bacterial, and environmental factors contributing to disease pathogenesis [1]. The factors that contribute to changes in the microbiome of CD patients are not well understood. Our findings demonstrating increased adhesiveness and biofilm formation of *E. coli* grown in MDX, but not invasion, would suggest that exposure to MDX may be one factor which promotes the colonization of pathobionts, such as AIEC LF82.

One factor which clearly influences the composition and characteristics of the microbiota is diet. Dietary studies in both mouse models and humans demonstrate large shifts in the composition of the microbiota dependent on diet [16], [25], [26], [27]. Comparisons of 16 S rRNA gene profiles between mice harboring a humanized microbiota and fed a high-fat/high-sugar diet (“Western diet”) versus those maintained on a low-fat/high polysaccharide diet revealed shifts in Bacteroidetes and an expansion of Bacilli and Erysipelotrichi [25]. Likewise, human studies comparing obese and lean twin pairs demonstrated changes in Bacteroidetes prevalence and a decrease in microbial diversity in obese individuals [47].

Diet also causes alterations in the types of genes present in microbial communities and influences their metabolism. Consumption of a Western diet enriches nutrient processing genes, such as ATP-binding cassette (ABC) transporters and phosphotransferase systems (PTS) [25], [47]. Interestingly, genes involved in MDX metabolism are in both these categories [48]. Notably, we show *malX* (a PTS gene of the maltose/MDX system) is more prevalent in the ileal mucosa-associated bacteria of CD patients. These findings suggest that MDX metabolism may be associated with the dysbiosis observed in CD and other diseases linked to Western diet consumption.

MDX is a d-glucose polymer under 20DE linked primarily by α(1→4) bonds produced through the enzymatic and chemical degradation of corn, potato or rice starch [29]. Since its introduction in the 1950 s, it has been added to a variety of foods and health care products to improve texture as a thickener, filler or binding agent. These MDX-containing products are diverse and include items such as non-calorie sweeteners (such as Splenda® and Equal®), snack foods, breakfast cereals, salad dressings, fiber supplements, hand lotions and medications. MDX is generally recognized as safe by the FDA (Code of Federal Regulations, Title 21, Volume 3, Part 184, Subpart B, Section 184.1444) and consumption of MDX is unlikely to be sufficient to cause disease in the absence of other risk factors. However, reports demonstrate that consumption of MDX or other polysaccharide additives under certain circumstances may result in intestinal disease [19], [20], [21], [22], [23], [24]. Specifically, studies associate induction of necrotizing enterocolitis in preterm piglets fed MDX-supplemented formula [20] and the production of diarrheal enterotoxin in infant milk-based formula made with MDX by *Bacillus cereus* [49]. Additionally, the ubiquitous inclusion of MDX into foods of the American diet parallels a substantial increase in incidence of CD [50], [51]. Our findings demonstrate that MDX enhances bacterial adhesion and suggests a mechanism by which consumption of this ubiquitous dietary additive may promote disease in susceptible individuals.

MDX is metabolized in the small intestine by specific enzymes [52], [53]. Interestingly, one of these enzymes (maltase-glucoamylase) is inhibited by high levels of MDX, suggesting that a MDX-rich diet could result in increased MDX levels in the small intestine and enrichment of MDX-utilizing bacteria in this location. In our studies, we observed MDX-enhanced biofilm formation by multiple strains of *E. coli* suggesting that MDX metabolism may be a dietary switch promoting colonization of these strains in new regions of the intestine (i.e. the ileum instead of the colon). Additionally, our data indicates that MDX-enhanced adhesion of LF82 is dependent on type 1 pili, but independent of the cellular receptor CEACAM6. This contrasts with studies defining CEACAM6 as a major cellular receptor for LF82 adhesion on CD patient enterocytes [38]. However, ileal epithelial expression of CEACAM6 is increased by LF82 infection, as well as pro-inflammatory cytokines. RNAi-mediated knockdown of CEACAM6 studies by Barnich et al., 2007 were performed with either stimulated Caco2 cells or CD patient-derived enterocytes and demonstrated a significant decrease in LF82 adhesion [38]. In our experiments, CEACAM6 knockdown did not affect LF82 adhesion to unstimulated Caco2 cells and this agrees with the authors' findings where no correlation between the basal levels of CEACAM6 expression in various cell lines and LF82 adhesion levels was observed. Of current discussion is whether the high levels of CEACAM6 expression seen in CD patients are present before disease onset (promoting disease susceptibility) or a consequence of disease pathogenesis (promoting disease progression) [38]. Our findings suggest a dietary mechanism by which LF82 could initially adhere to the epithelium and then induce the expression of CEACAM6 as a secondary event to maintain its colonization of the ileum and promote disease.

Finally, although there is a strong link between AIEC strains and CD pathogenesis, no one bacterial strain has been causatively linked to CD [3]. Other candidates include *Mycobacterium avium* subspecies *paratuberculosis*, *Yersinia enterocolitica*, *Listeria monocytogenes*, *Salmonella* spp., *Clostridium difficile*, *Enterococcus feacalis,* and *Campylobacter* spp [54]. Interestingly, several of these disease-associated strains possess the *malX* gene or have homologous systems for MDX utilization (Figure S3). This observation suggests that MDX metabolism may promote colonization of multiple CD-associated bacteria and is supported by our findings that *malX* prevalence is significantly increased in ileal CD patient mucosa in the absence of increased *E. coli* colonization. This leads us to postulate that consumption of MDX could increase bacterial loads in the ileum and prime these individuals to have a greater translocation of bacteria after intestinal injury. If these individuals carry other risk factors for CD (genetic variants of anti-bacterial response genes such as *ATG16L1* or *NOD2*, for example), this may result in the development of disease in these susceptible individuals. These findings describe a potential disease mechanism linking the ubiquitous dietary additive MDX to microbial changes in the intestine of CD patients and suggest a novel therapeutic area for the prevention and treatment of inflammatory bowel disease.

## Supporting Information

Figure S1 **MDX included as a bulking agent in non-calorie sweeteners enhances biofilm formation.** (A) Growth of LF82 in medium supplemented with the indicated sweetener. (B) Specific biofilm formation of LF82. Average ±SD shown. *p<0.05, **p<0.01 (C) Micrographs of biofilms from B stained with either crystal violet to detect adhered bacteria or Congo red to visualize the exopolysaccharide matrix. (D) Effect of aspartame or sucralose on biofilm formation of LF82. Average ±SD shown. **p<0.01 (E) Micrographs of crystal violet stained biofilms from D.
(TIF)

Figure S2 **Effect of different MDX chain lengths on LF82 growth and biofilm formation.** (A) Growth curves of LF82 in M9 medium supplemented with the indicated sugar. (B) Micrographs of Congo red stained LF82 biofilms formed in medium supplemented with the indicated sugar for 24 h.
(TIF)

Figure S3 **Identification strategy for** ***malX*** **^+^ bacterial strains to be analyzed by quantitative PCR in human mucosal samples.** NCBI lists 63 proteobacteria species with gene sequences specific for *malX*. Excluding discontinued sequences and partial sequences, the remaining sequences were aligned using ClustlW2. Further sequences were eliminated if they lacked significant identity or were sequences from non-human pathogens for a final strain count of 36 gene sequences. The aligned sequence was used to generate a consensuses sequence which was then entered into the BiBiServ GeneFisher2 software. Parameters for primer design were length between 15 to 18 bp, GC content of 45–65%, melting temperature between 57–63°C and a product size between 50 and 200bp. Candidate primer sets were also evaluated for possible amplification of the human genome. The primers selected were 5′ACGCGTTTCCTTTCGCAA3′ and 5′ACAGAACTGGCGCTACGA3′.
(TIF)

Table S1 **Characteristics of Tissue Donors.**
(DOC)

## References

[1] Cosnes J, Gower-Rousseau C, Seksik P, Cortot A (2011) Epidemiology and natural history of inflammatory bowel diseases. Gastroenterology 140: 1785–1794. 21530745 10.1053/j.gastro.2011.01.055

[2] Cho JH, Brant SR (2011) Recent insights into the genetics of inflammatory bowel disease. Gastroenterology 140: 1704–1712. 21530736 10.1053/j.gastro.2011.02.046 PMC4947143

[3] Chassaing B, Darfeuille-Michaud A (2011) The commensal microbiota and enteropathogens in the pathogenesis of inflammatory bowel diseases. Gastroenterology 140: 1720–1728. 21530738 10.1053/j.gastro.2011.01.054

[4] Manichanh C, Rigottier-Gois L, Bonnaud E, Gloux K, Pelletier E, et al (2006) Reduced diversity of faecal microbiota in Crohn's disease revealed by a metagenomic approach. Gut 55: 205–211. 16188921 10.1136/gut.2005.073817 PMC1856500

[5] Cotter PD (2011) Small intestine and microbiota. Curr Opin Gastroenterol 27: 99–105. 21102323 10.1097/MOG.0b013e328341dc67

[6] Martinez-Medina M, Aldeguer X, Gonzalez-Huix F, Acero D, Garcia-Gil LJ (2006) Abnormal microbiota composition in the ileocolonic mucosa of Crohn's disease patients as revealed by polymerase chain reaction-denaturing gradient gel electrophoresis. Inflamm Bowel Dis 12: 1136–1145. 17119388 10.1097/01.mib.0000235828.09305.0c

[7] Keighley MR, Arabi Y, Dimock F, Burdon DW, Allan RN, et al (1978) Influence of inflammatory bowel disease on intestinal microflora. Gut 19: 1099–1104. 369959 10.1136/gut.19.12.1099 PMC1412333

[8] Sokol H, Seksik P, Furet JP, Firmesse O, Nion-Larmurier I, et al (2009) Low counts of Faecalibacterium prausnitzii in colitis microbiota. Inflamm Bowel Dis 15: 1183–1189. 19235886 10.1002/ibd.20903

[9] Baker PI, Love DR, Ferguson LR (2009) Role of gut microbiota in Crohn's disease. Expert Rev Gastroenterol Hepatol 3: 535–546. 19817674 10.1586/egh.09.47

[10] Boudeau J, Glasser AL, Masseret E, Joly B, Darfeuille-Michaud A (1999) Invasive ability of an Escherichia coli strain isolated from the ileal mucosa of a patient with Crohn's disease. Infect Immun 67: 4499–4509. 10456892 10.1128/iai.67.9.4499-4509.1999 PMC96770

[11] Darfeuille-Michaud A, Neut C, Barnich N, Lederman E, Di Martino P, et al (1998) Presence of adherent Escherichia coli strains in ileal mucosa of patients with Crohn's disease. Gastroenterology 115: 1405–1413. 9834268 10.1016/s0016-5085(98)70019-8

[12] Vejborg RM, Hancock V, Petersen AM, Krogfelt KA, Klemm P (2011) Comparative genomics of Escherichia coli isolated from patients with inflammatory bowel disease. BMC Genomics 12: 316. 21676223 10.1186/1471-2164-12-316 PMC3155842

[13] Vasquez N, Mangin I, Lepage P, Seksik P, Duong JP, et al (2007) Patchy distribution of mucosal lesions in ileal Crohn's disease is not linked to differences in the dominant mucosa-associated bacteria: a study using fluorescence in situ hybridization and temporal temperature gradient gel electrophoresis. Inflamm Bowel Dis 13: 684–692. 17206669 10.1002/ibd.20084

[14] Swidsinski A, Loening-Baucke V, Herber A (2009) Mucosal flora in Crohn's disease and ulcerative colitis – an overview. J Physiol Pharmacol 60 Suppl 661–71. 20224153

[15] Swidsinski A, Weber J, Loening-Baucke V, Hale LP, Lochs H (2005) Spatial organization and composition of the mucosal flora in patients with inflammatory bowel disease. J Clin Microbiol 43: 3380–3389. 16000463 10.1128/JCM.43.7.3380-3389.2005 PMC1169142

[16] Spor A, Koren O, Ley R (2011) Unravelling the effects of the environment and host genotype on the gut microbiome. Nat Rev Microbiol 9: 279–290. 21407244 10.1038/nrmicro2540

[17] Stowe SP, Redmond SR, Stormont JM, Shah AN, Chessin LN, et al (1990) An epidemiologic study of inflammatory bowel disease in Rochester, New York. Hospital incidence. Gastroenterology 98: 104–110. 2293567 10.1016/0016-5085(90)91297-j

[18] BeMiller JN (2009) One hundred years of commercial food carbohydrates in the United States. J Agric Food Chem 57: 8125–8129. 19719134 10.1021/jf8039236

[19] Swidsinski A, Ung V, Sydora BC, Loening-Baucke V, Doerffel Y, et al (2009) Bacterial overgrowth and inflammation of small intestine after carboxymethylcellulose ingestion in genetically susceptible mice. Inflamm Bowel Dis 15: 359–364. 18844217 10.1002/ibd.20763

[20] Thymann T, Moller HK, Stoll B, Stoy AC, Buddington RK, et al (2009) Carbohydrate maldigestion induces necrotizing enterocolitis in preterm pigs. Am J Physiol Gastrointest Liver Physiol 297: G1115–1125. 19808655 10.1152/ajpgi.00261.2009 PMC2850085

[21] Beal J, Silverman B, Bellant J, Young TE, Klontz K (2012) Late Onset Necrotizing Enterocolitis in Infants following Use of a Xanthan Gum-Containing Thickening Agent. J Pediatr. 10.1016/j.jpeds.2012.03.054 22575248

[22] Tobacman JK (2001) Review of harmful gastrointestinal effects of carrageenan in animal experiments. Environ Health Perspect 109: 983–994. 11675262 10.1289/ehp.01109983 PMC1242073

[23] Montagne L, Cavaney FS, Hampson DJ, Lalles JP, Pluske JR (2004) Effect of diet composition on postweaning colibacillosis in piglets. J Anim Sci 82: 2364–2374. 15318736 10.2527/2004.8282364x

[24] McDonald DE, Pethick DW, Mullan BP, Hampson DJ (2001) Increasing viscosity of the intestinal contents alters small intestinal structure and intestinal growth, and stimulates proliferation of enterotoxigenic Escherichia coli in newly-weaned pigs. Br J Nutr 86: 487–498. 11591236 10.1079/bjn2001416

[25] Turnbaugh PJ, Ridaura VK, Faith JJ, Rey FE, Knight R, et al (2009) The effect of diet on the human gut microbiome: a metagenomic analysis in humanized gnotobiotic mice. Sci Transl Med 1: 6ra14. 10.1126/scitranslmed.3000322 PMC2894525 20368178

[26] Muegge BD, Kuczynski J, Knights D, Clemente JC, Gonzalez A, et al (2011) Diet drives convergence in gut microbiome functions across mammalian phylogeny and within humans. Science 332: 970–974. 21596990 10.1126/science.1198719 PMC3303602

[27] Wu GD, Chen J, Hoffmann C, Bittinger K, Chen YY, et al (2011) Linking long-term dietary patterns with gut microbial enterotypes. Science 334: 105–108. 21885731 10.1126/science.1208344 PMC3368382

[28] Hehemann JH, Correc G, Barbeyron T, Helbert W, Czjzek M, et al (2010) Transfer of carbohydrate-active enzymes from marine bacteria to Japanese gut microbiota. Nature 464: 908–912. 20376150 10.1038/nature08937

[29] Chronakis IS (1998) On the molecular characteristics, compositional properties, and structural-functional mechanisms of maltodextrins: a review. Crit Rev Food Sci Nutr 38: 599–637. 9813736 10.1080/10408699891274327

[30] Jensen SR, Fink LN, Struve C, Sternberg C, Andersen JB, et al (2011) Quantification of specific E. coli in gut mucosa from Crohn's disease patients. J Microbiol Methods 86: 111–114. 21504765 10.1016/j.mimet.2011.04.002

[31] Nadkarni MA, Martin FE, Jacques NA, Hunter N (2002) Determination of bacterial load by real-time PCR using a broad-range (universal) probe and primers set. Microbiology 148: 257–266. 11782518 10.1099/00221287-148-1-257

[32] Djordjevic D, Wiedmann M, McLandsborough LA (2002) Microtiter plate assay for assessment of Listeria monocytogenes biofilm formation. Appl Environ Microbiol 68: 2950–2958. 12039754 10.1128/AEM.68.6.2950-2958.2002 PMC123944

[33] Schwan WR, Seifert HS, Duncan JL (1992) Growth conditions mediate differential transcription of fim genes involved in phase variation of type 1 pili. J Bacteriol 174: 2367–2375. 1348054 10.1128/jb.174.7.2367-2375.1992 PMC205860

[34] Boos W, Shuman H (1998) Maltose/maltodextrin system of Escherichia coli: transport, metabolism, and regulation. Microbiol Mol Biol Rev 62: 204–229. 9529892 10.1128/mmbr.62.1.204-229.1998 PMC98911

[35] Hill DR, Kessler SP, Rho HK, Cowman MK, de la Motte CA (2012) Specific-sized hylauronan fragments promote expression of human beta-defensin 2 in intestinal epithelium. Journal of Biological Chemistry. 10.1074/jbc.M112.356238 PMC3436307 22761444

[36] Rolhion N, Carvalho FA, Darfeuille-Michaud A (2007) OmpC and the sigma(E) regulatory pathway are involved in adhesion and invasion of the Crohn's disease-associated Escherichia coli strain LF82. Mol Microbiol 63: 1684–1700. 17367388 10.1111/j.1365-2958.2007.05638.x

[37] Prigent-Combaret C, Brombacher E, Vidal O, Ambert A, Lejeune P, et al (2001) Complex regulatory network controls initial adhesion and biofilm formation in Escherichia coli via regulation of the csgD gene. J Bacteriol 183: 7213–7223. 11717281 10.1128/JB.183.24.7213-7223.2001 PMC95571

[38] Barnich N, Carvalho FA, Glasser AL, Darcha C, Jantscheff P, et al (2007) CEACAM6 acts as a receptor for adherent-invasive E. coli, supporting ileal mucosa colonization in Crohn disease. J Clin Invest 117: 1566–1574. 17525800 10.1172/JCI30504 PMC1868786

[39] Claret L, Miquel S, Vieille N, Ryjenkov DA, Gomelsky M, et al (2007) The flagellar sigma factor FliA regulates adhesion and invasion of Crohn disease-associated Escherichia coli via a cyclic dimeric GMP-dependent pathway. J Biol Chem 282: 33275–33283. 17827157 10.1074/jbc.M702800200

[40] Carvalho FA, Barnich N, Sauvanet P, Darcha C, Gelot A, et al (2008) Crohn's disease-associated Escherichia coli LF82 aggravates colitis in injured mouse colon via signaling by flagellin. Inflamm Bowel Dis 14: 1051–1060. 18338780 10.1002/ibd.20423

[41] Sepehri S, Khafipour E, Bernstein CN, Coombes BK, Pilar AV, et al (2011) Characterization of Escherichia coli isolated from gut biopsies of newly diagnosed patients with inflammatory bowel disease. Inflamm Bowel Dis 17: 1451–1463. 21674703 10.1002/ibd.21509

[42] Abbott DW, Higgins MA, Hyrnuik S, Pluvinage B, Lammerts van Bueren A, et al (2010) The molecular basis of glycogen breakdown and transport in Streptococcus pneumoniae. Mol Microbiol 77: 183–199. 20497336 10.1111/j.1365-2958.2010.07199.x PMC2911477

[43] Swidsinski A, Ladhoff A, Pernthaler A, Swidsinski S, Loening-Baucke V, et al (2002) Mucosal flora in inflammatory bowel disease. Gastroenterology 122: 44–54. 11781279 10.1053/gast.2002.30294

[44] Kau AL, Ahern PP, Griffin NW, Goodman AL, Gordon JI (2011) Human nutrition, the gut microbiome and the immune system. Nature 474: 327–336. 21677749 10.1038/nature10213 PMC3298082

[45] Sekirov I, Russell SL, Antunes LC, Finlay BB (2010) Gut microbiota in health and disease. Physiol Rev 90: 859–904. 20664075 10.1152/physrev.00045.2009

[46] Sansonetti PJ (2011) To be or not to be a pathogen: that is the mucosally relevant question. Mucosal Immunol 4: 8–14. 21150896 10.1038/mi.2010.77

[47] Turnbaugh PJ, Hamady M, Yatsunenko T, Cantarel BL, Duncan A, et al (2009) A core gut microbiome in obese and lean twins. Nature 457: 480–484. 19043404 10.1038/nature07540 PMC2677729

[48] Reidl J, Boos W (1991) The malX malY operon of Escherichia coli encodes a novel enzyme II of the phosphotransferase system recognizing glucose and maltose and an enzyme abolishing the endogenous induction of the maltose system. J Bacteriol 173: 4862–4876. 1856179 10.1128/jb.173.15.4862-4876.1991 PMC208166

[49] Rowan NJ, Anderson JG (1997) Maltodextrin stimulates growth of Bacillus cereus and synthesis of diarrheal enterotoxin in infant milk formulae. Appl Environ Microbiol 63: 1182–1184. 9055435 10.1128/aem.63.3.1182-1184.1997 PMC168410

[50] Loftus EV (2004) Clinical epidemiology of inflammatory bowel disease: Incidence, prevalence, and environmental influences. Gastroenterology 126: 1504–1517. 15168363 10.1053/j.gastro.2004.01.063

[51] Gross LS, Li L, Ford ES, Liu S (2004) Increased consumption of refined carbohydrates and the epidemic of type 2 diabetes in the United States: an ecologic assessment. Am J Clin Nutr 79: 774–779. 15113714 10.1093/ajcn/79.5.774

[52] Quezada-Calvillo R, Robayo-Torres CC, Ao Z, Hamaker BR, Quaroni A, et al (2007) Luminal substrate “brake” on mucosal maltase-glucoamylase activity regulates total rate of starch digestion to glucose. J Pediatr Gastroenterol Nutr 45: 32–43. 17592362 10.1097/MPG.0b013e31804216fc

[53] Engfer MB, Stahl B, Finke B, Sawatzki G, Daniel H (2000) Human milk oligosaccharides are resistant to enzymatic hydrolysis in the upper gastrointestinal tract. Am J Clin Nutr 71: 1589–1596. 10837303 10.1093/ajcn/71.6.1589

[54] Kalischuk LD, Buret AG (2009) A role for Campylobacter jejuni-induced enteritis in inflammatory bowel disease? Am J Physiol Gastrointest Liver Physiol 298: G1–9. 19875702 10.1152/ajpgi.00193.2009

[55] Martinez-Medina M, Aldeguer X, Lopez-Siles M, Gonzalez-Huix F, Lopez-Oliu C, et al (2009) Molecular diversity of Escherichia coli in the human gut: new ecological evidence supporting the role of adherent-invasive E. coli (AIEC) in Crohn's disease. Inflamm Bowel Dis 15: 872–882. 19235912 10.1002/ibd.20860

[56] Martinez-Medina M, Naves P, Blanco J, Aldeguer X, Blanco JE, et al (2009) Biofilm formation as a novel phenotypic feature of adherent-invasive Escherichia coli (AIEC). BMC Microbiol 9: 202. 19772580 10.1186/1471-2180-9-202 PMC2759958

[57] Barnich N, Boudeau J, Claret L, Darfeuille-Michaud A (2003) Regulatory and functional co-operation of flagella and type 1 pili in adhesive and invasive abilities of AIEC strain LF82 isolated from a patient with Crohn's disease. Mol Microbiol 48: 781–794. 12694621 10.1046/j.1365-2958.2003.03468.x

[58] Simonsen KT, Nielsen G, Bjerrum JV, Kruse T, Kallipolitis BH, et al (2011) A role for the RNA chaperone Hfq in controlling adherent-invasive Escherichia coli colonization and virulence. PLoS One 6: e16387. 21298102 10.1371/journal.pone.0016387 PMC3027648

