# Supplementary figures and images for: Crohn's Disease-Associated Adherent-Invasive Escherichia coli Adhesion Is Enhanced by Exposure to the Ubiquitous Dietary Polysaccharide Maltodextrin
Source: PLoS One. 2012 Dec 12;7(12):e52132. doi: 10.1371/journal.pone.0052132 (PMC3520894; doi:10.1371/journal.pone.0052132)

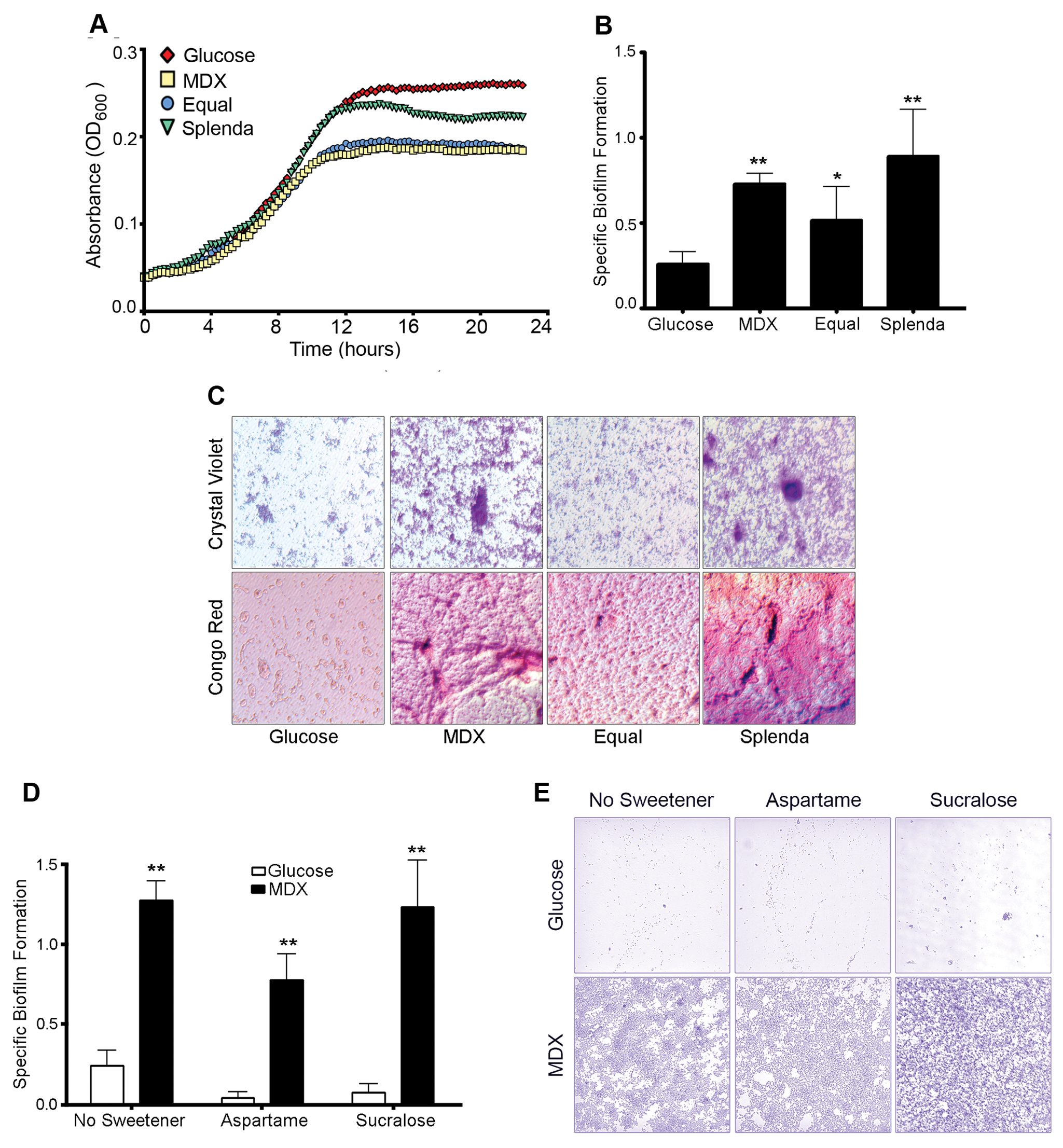

Supplement: Figure S1 — MDX included as a bulking agent in non-calorie sweeteners enhances biofilm formation. (A) Growth of LF82 in medium supplemented with the indicated sweetener. (B) Specific biofilm formation of LF82. Average ±SD shown. *p<0.05, **p<0.01 (C) Micrographs of biofilms from B stained with either crystal violet to detect adhered bacteria or Congo red to visualize the exopolysaccharide matrix. (D) Effect of aspartame or sucralose on biofilm formation of LF82. Average ±SD shown. **p<0.01 (E) Micrographs of crystal violet stained biofilms from D. (TIF) [file pone.0052132.s001.tif]

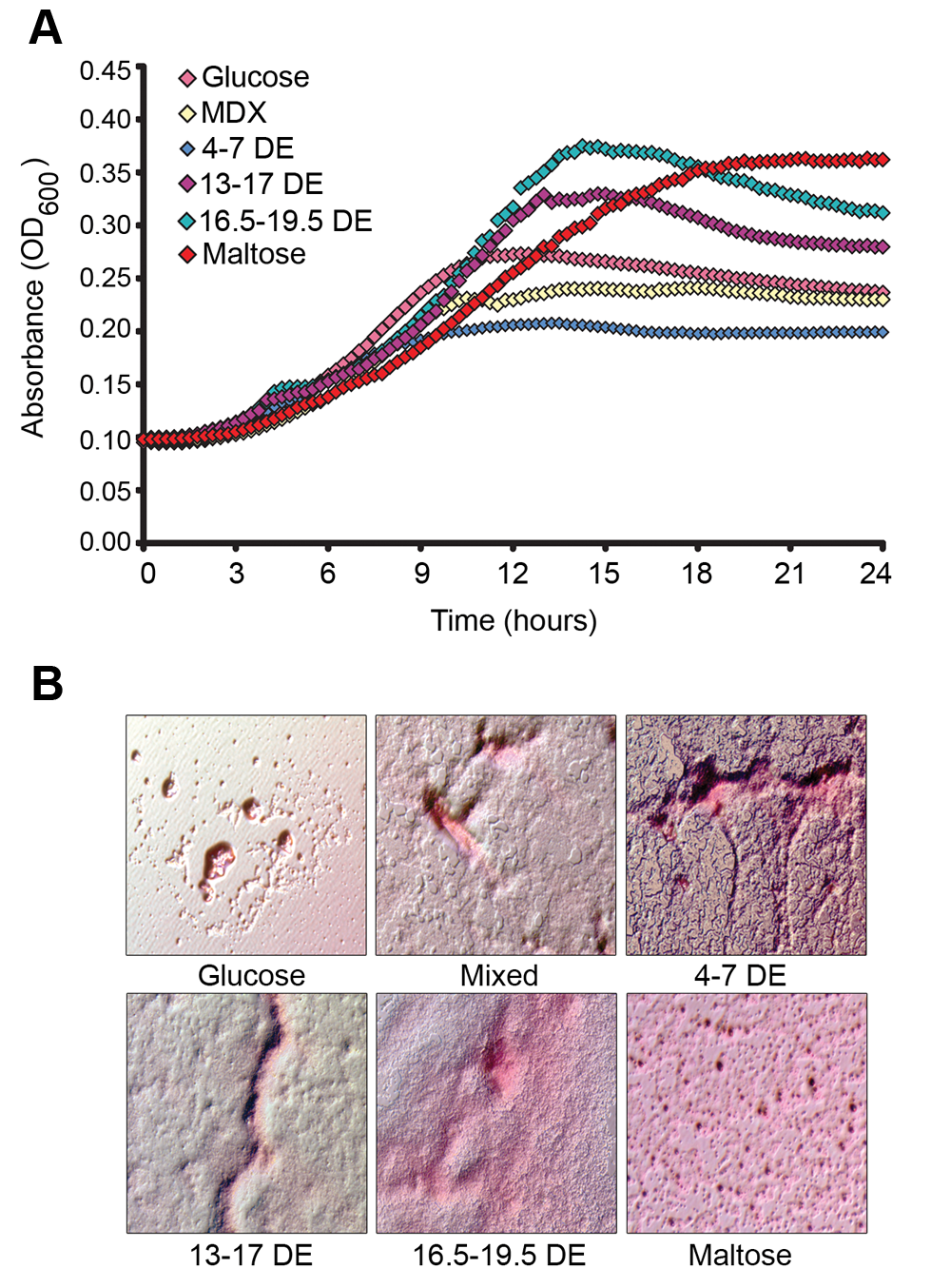

Supplement: Figure S2 — Effect of different MDX chain lengths on LF82 growth and biofilm formation. (A) Growth curves of LF82 in M9 medium supplemented with the indicated sugar. (B) Micrographs of Congo red stained LF82 biofilms formed in medium supplemented with the indicated sugar for 24 h. (TIF) [file pone.0052132.s002.tif]

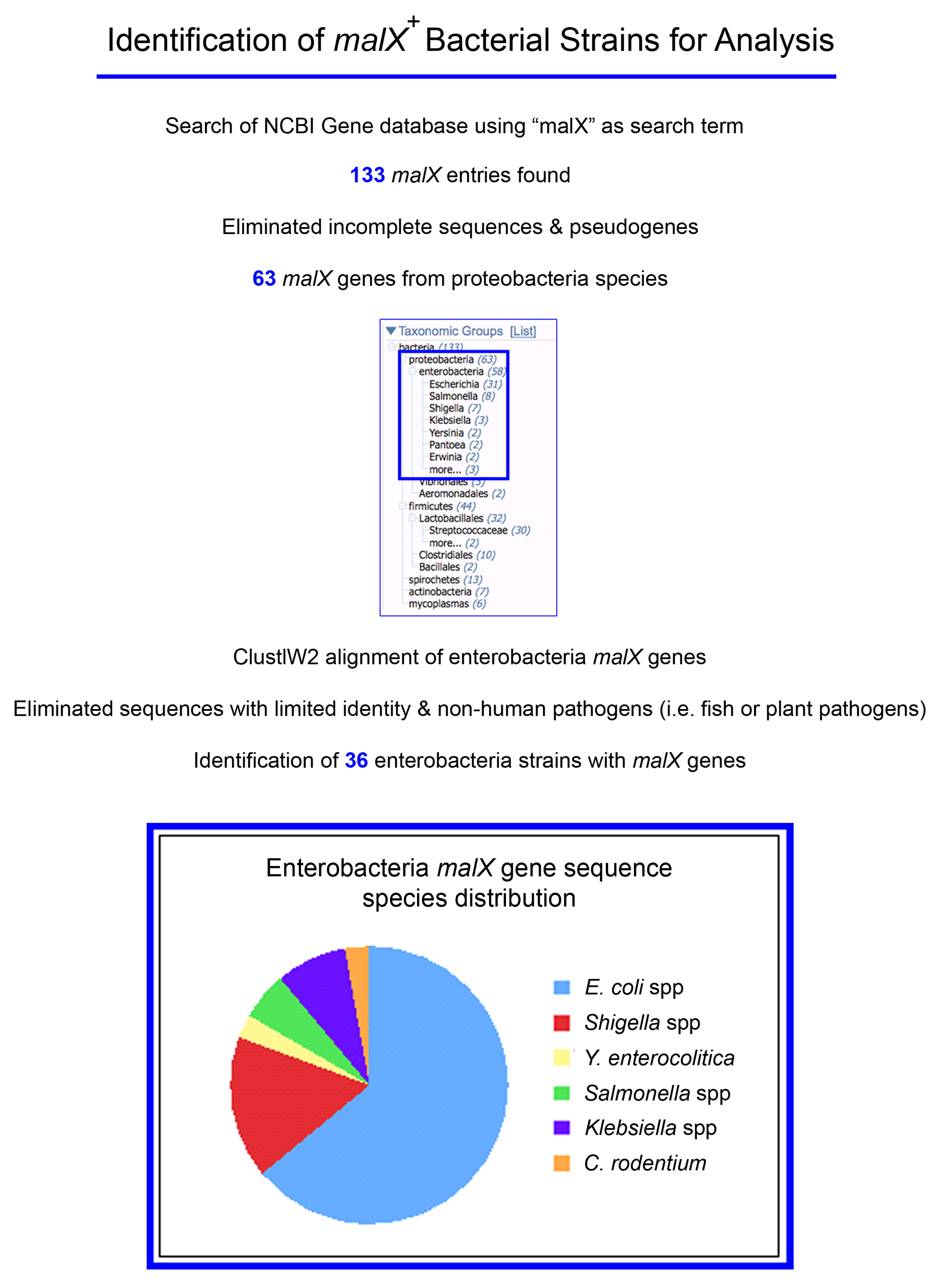

Supplement: Figure S3 — Identification strategy for malX + bacterial strains to be analyzed by quantitative PCR in human mucosal samples. NCBI lists 63 proteobacteria species with gene sequences specific for malX. Excluding discontinued sequences and partial sequences, the remaining sequences were aligned using ClustlW2. Further sequences were eliminated if they lacked significant identity or were sequences from non-human pathogens for a final strain count of 36 gene sequences. The aligned sequence was used to generate a consensuses sequence which was then entered into the BiBiServ GeneFisher2 software. Parameters for primer design were length between 15 to 18 bp, GC content of 45–65%, melting temperature between 57–63°C and a product size between 50 and 200bp. Candidate primer sets were also evaluated for possible amplification of the human genome. The primers selected were 5′ACGCGTTTCCTTTCGCAA3′ and 5′ACAGAACTGGCGCTACGA3′. (TIF) [file pone.0052132.s003.tif]
