# Supplementary material for: Crohn's Disease-Associated Adherent-Invasive Escherichia coli Adhesion Is Enhanced by Exposure to the Ubiquitous Dietary Polysaccharide Maltodextrin
Source: PLoS One. 2012 Dec 12;7(12):e52132. doi: 10.1371/journal.pone.0052132 (PMC3520894; doi:10.1371/journal.pone.0052132)
Supplement: Table S1 — Characteristics of Tissue Donors. (DOC) [file pone.0052132.s004.doc]

**Supplemental Table** 1: Characteristics of Tissue Donors

| **Experimental Group** | **Tissue** | **Median Age (Range)** | **Gender (F/M)** | **Diagnoses** |
| --- | --- | --- | --- | --- |
| Controls | Ileum | 50 (24-78) | 6/4 | Colon cancer, familial adenomatous polyposis, tumor, diverticular disease |
| CD | Ileum | 34.9 (17-76) | 9/9 | Crohn’s Disease |
| CD | Colon | 32 (22-67) | 8/8 | Crohn’s Disease |
